# “Before Xpert I only had my expertise”: A qualitative study on the utilization and effects of Xpert technology among pediatricians in 4 Indian cities

**DOI:** 10.1371/journal.pone.0193656

**Published:** 2018-03-16

**Authors:** Andrew McDowell, Neeraj Raizada, Sunil D. Khaparde, Raghuram Rao, Sanjay Sarin, Aakshi Kalra, Virender Singh Salhotra, Sreenivas Achuthan Nair, Catharina Boehme, Claudia M. Denkinger

**Affiliations:** 1 Institut National de la Santé et Recherché Medical, Paris, France; 2 Harvard Medical School Center for Global Health Delivery, Dubai, United Arab Emirates; 3 Foundation for Innovative New Diagnostics, New Delhi, India; 4 Central TB Division, Government of India, New Delhi, India; 5 World Health Organization, Country Office for India, New Delhi, India; 6 Foundation for Innovative New Diagnostics, Geneva, Switzerland; Médecins Sans Frontières (MSF), SOUTH AFRICA

## Abstract

**Background:**

Diagnosing tuberculosis (TB) in children presents considerable challenges. Upfront testing on Xpert® MTB/RIF (‘Xpert’)—a rapid molecular assay with high sensitivity and specificity—for pediatric presumptive TB patients, as recommended by India’s Revised National Tuberculosis Control Program (RNTCP), can pave the way for early TB diagnosis. As part of an ongoing project implemented by Foundation for Innovative New Diagnostics (FIND) dedicated to providing upfront free-of-cost (FOC) Xpert testing to children seeking care in the public and private sectors, a qualitative assessment was designed to understand how national guidelines on TB diagnosis and Xpert technology have been integrated into the pediatric TB care practices of different health providers.

**Methods:**

We conducted semi-structured interviews with a sample of health providers from public and private sectors engaged in the ongoing pediatric project in 4 major cities of India. Providers were sampled from intervention data based on sector of practice, number of Xpert referrals, and TB detection rates amongst referrals. A total of 55 providers were interviewed with different levels of FOC Xpert testing uptake. Data were transcribed and analyzed inductively by a medical anthropologist using thematic content analysis and narrative analysis.

**Results:**

It was observed that despite guidance from RNTCP on the use of Xpert and significant efforts by FIND and state authorities to disseminate these guidelines, there was notable diversity in their implementation by different health care providers. Xpert, apart from being utilized as intended, i.e. as a first diagnostic test for children, was utilized variably–as an initial screening test (to rule out TB), confirmatory test (once TB diagnosis is established based on antibiotic trial or clinically) and/or only for drug susceptibility testing after TB diagnosis was confirmed. Most providers who used Xpert frequently reported that Xpert was an important tool for managing pediatric TB cases, by reducing the proportion of cases diagnosed only on clinical suspicion and by providing upfront information on drug resistance, which is seldom suspected in children. Despite non-standard use, these results showed that Xpert access helped raise awareness, aided in antibiotic stewardship, and reduced dependence on clinical diagnosis among those who diagnose and treat TB in children.

**Conclusion:**

Access to free and rapid Xpert testing for all presumptive pediatric TB patients has had multiple positive effects on pediatricians’ diagnosis and treatment of TB. It has important effects on speed of diagnosis, empirical treatment, and awareness of drug resistance among TB treatment naive children. In addition, our study shows that access to public sector Xpert machines may be an important way to encourage Public-Private integration and facilitate the movement of patients from the private to public sector for anti-TB treatment. Despite availability of rapid and free Xpert testing, our study showed an alarming diversity of Xpert utilization strategies across different providers who may be moving toward suggested practice over time. The degree of diversity in TB diagnostic approaches in children reported here highlights the urgent need for concerted efforts to place Xpert early in diagnostic algorithms to positively impact the pediatric TB care pathway. A positive change in diagnostic algorithms may be possible with continued advocacy, time, and increased access.

## Introduction

India is home to the largest number of people suffering from TB in the world [1] and about 6% of all notified TB cases in India in 2014 were children [2]. Despite declines in the annual risk of TB infection in India[3], pediatric TB is a significant concern associated with substantial morbidity and mortality [4, 5].

The obstacles to diagnosing TB in children are many and include difficult access to high quality samples, rapid disease progression, and higher rates of extra-pulmonary infection [5]. Clinical diagnosis of TB can be challenging, as signs and symptoms of TB in children can be non-specific and similar to other common childhood chest infections [5, 6]. Diagnostic efforts are also undermined by the lack of diagnostic tests with high sensitivity that are simple to use and can be applied at the point of care [7]. Isolation of *M*. *tuberculosis* by culture, while considered a gold standard, requires long turnaround time and related infrastructural costs, which make it impractical to use in routine paediatric TB case management [8, 9]. Drug resistance in children is almost never evaluated, which limits the identification of rifampicin-resistant TB (RR-TB) [10]. Due to these challenges, diagnosis of TB in children is largely based on a history of contact with a TB patient, clinical and radiological findings, and often without microbiological confirmation[11, 12].

**Fig 1:**
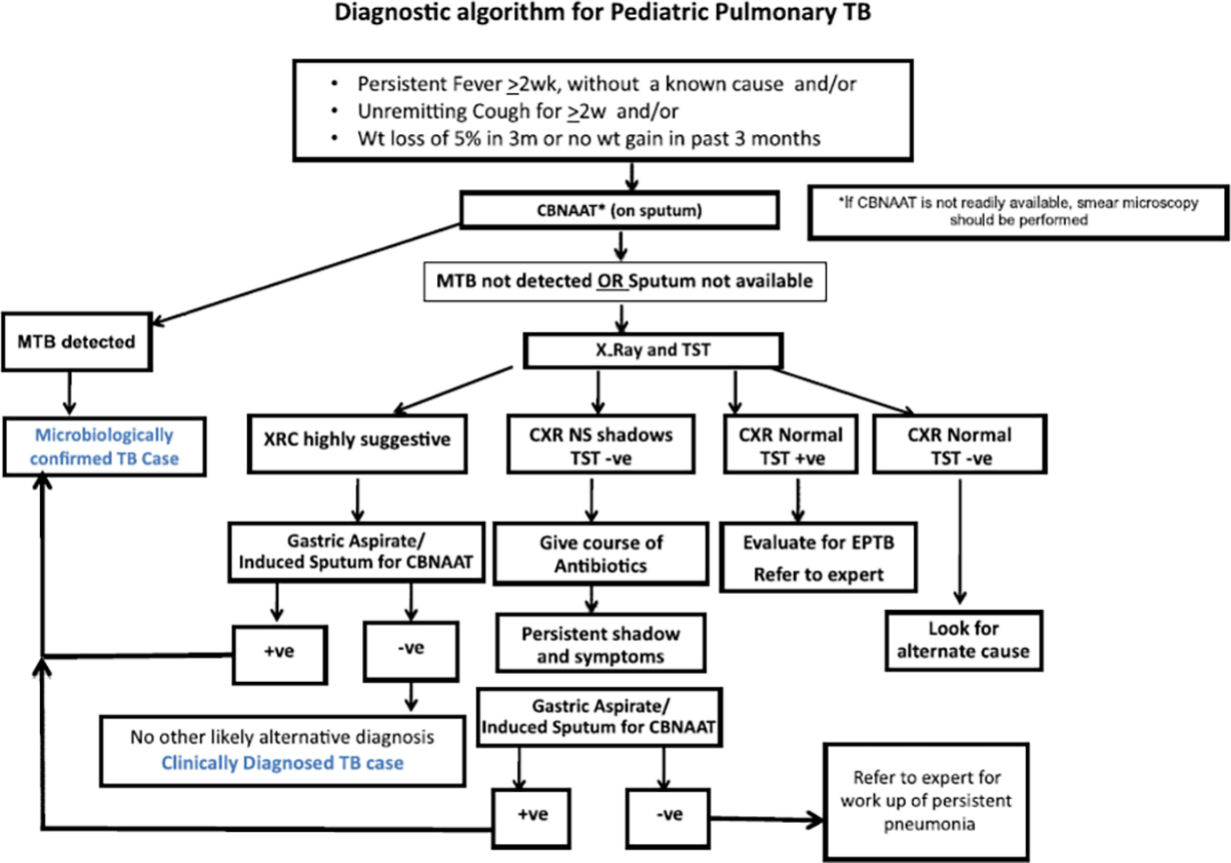
RNTCP policy recommendation for the diagnosis of pediatric TB as per the technical and operational guidelines for TB control in India, 2016.

Xpert MTB/RIF (‘Xpert’) can be of particular benefit for diagnosing TB in pediatric populations because of (i) its higher sensitivity in comparison to smear microscopy [10] and (ii) its ability to rapidly provide a result. Since the WHO endorsement of Xpert in 2010 [13], its introduction in India in 2012 [14], and the WHO revised policy statement suggesting the use of Xpert for all adults and children presumed to have TB [15], Xpert use and availability in India has increased rapidly[16]. Xpert is offered free of cost in the public sector and at a reduced and controlled price in parts of the private sector through various initiatives [17]. According to Revised National TB Control Program (RNTCP) protocols, all pediatric presumptive TB cases should be offered upfront Xpert testing [18](Fig 1).

The Foundation for Innovation in New Diagnostics (FIND) in collaboration with the RNTCP undertook an innovative pediatric project in Chennai, Delhi, Hyderabad, and Kolkata to catalyze the effective implementation of the national policy of upfront Xpert for pediatric presumptive TB cases, both in the public and private sectors [10]. The project offered free testing with a quick turnaround time and also included several efforts in coordination with state authorities and local medical bodies in these cities to improve provider literacy with respect to diagnosis of TB in children and improve the project uptake.

As a part of this project, a four-city qualitative study of TB treating physicians was designed to assess Xpert prescription practices and its perceived diagnostic utility across sectors as well as understand barriers to Xpert use in pediatric populations. We aimed to answer three research questions: i) how do pediatricians use Xpert when accessible and free of cost, ii) how do they prioritize and evaluate Xpert in relation to other diagnostic technologies, and iii) what are the effects of Xpert on their clinical practice.

## Method

### Intervention context

The current study was undertaken to evaluate and inform a USAID-funded Challenge TB project implemented in 4 major cities of India from April 2014 to June 2016, namely Chennai, Delhi, Hyderabad and Kolkata, covering a population of more than 30 million, with the objective of providing all pediatric presumptive TB patients in these cities upfront free-of-charge access to Xpert testing. Under the ongoing project, high throughput Xpert laboratories were established—dedicated to the pediatric population. Using a hub and spoke model, one laboratory was established in each city which linked to various public and private sector health care providers. Samples were collect in clinics and hospitals by physicians or nurses, or patient’s home in the case of expectorated sputum, using a range of methodologies. Samples included gastric lavage/aspirate (GA), broncheoalveolar lavage (BAL), expectorated and induced sputum, lymph node aspirates, cerebrospinal fluid (CSF), and pus among others [10]. Specimen transportation linkages were optimized, taking into account feasible local transportation mechanisms acceptable to various health facilities and providers, such as commercial courier services and local volunteers whose incidental costs were reimbursed at a standard rate. A rapid reporting mechanism was established to ensure that all test results were promptly communicated back to providers utilizing e-mail and short messaging service (SMS). Health care providers (both public and private) in the project cities were identified through detailed mapping. The providers were encouraged to prescribe upfront free-of-charge Xpert testing for pediatric presumptive TB patients that presented to their health facilities. Several sensitization workshops and clinic visits by project staff who interacted with providers through the Xpert process were undertaken with pediatric health providers to increase the project uptake. The current project represents one of largest global efforts exclusively dedicated to the implementation of WHO guidelines on the use of upfront Xpert testing for the pediatric population.

### Qualitative study context

The study used semi-structured interviewing to understand the effects of a global health intervention on Xpert use in urban India for the diagnosis on TB in children. It included equal numbers of private and public sector physicians in the four cities where FOC Xpert facilities were made available, fully dedicated to pediatric population. The interviews were conducted after nearly two years of initiation of the intervention. Only physicians who had referred at least one children for TB screening under the project were interviewed. The study provided anonymized feedback to intervention partners and contributes to the knowledge of pediatric TB care in India’s public and private sector clinics.

### Study design, setting, and sampling

We designed a qualitative study of physicians engaged under the ongoing project, who referred presumptive pediatric TB patients for Xpert testing. The study relied on semi-structured interviews by a qualitative researcher. The study was designed to better understand the perspective of providers engaged under the ongoing project with respect to Xpert testing, related national and global guidance for the diagnosis of TB in children, and various bottlenecks in its effective implementation. The main purpose of the study was to inform the RNTCP’s efforts to improve pediatric TB care. The selection of 4 project cities was purposive. These 4 cities, among India’s most populous, were selected to maximize the impact and uptake of the project interventions, and due to the absence of other similar initiatives. These cities are known to have a concentration of public and private sector pediatricians. Interviews were conducted in clinics, hospitals, and medical colleges.

Physicians selection for interview was based on systematic puroposive sampling to include public and private providers equally while capturing diversity in quantum of referrals for Xpert testing and TB positivity in their settings. We used program data to identify the top, middle, and bottom third of Xpert referrers in each city. Next we divided the physicians into two groups, based on TB detection rates. Finally, we randomly selected one public and one privately practicing provider from each group. In each city we interviewed one high and one low positivity rate user from each of the three groups of referrers. If sampled physicians were unavailable we sampled again from the same group. The sampling procedure was identical for all four cities. All interviewees were medical doctors and all had 5 or more years of clinical experience.

### Data collection

Data were collected in April 2016 using qualitative semi-structured in-depth interview guides. Over time we added new topics to the lists but did not remove any existing topics in the lists. To facilitate narrative coherence and build rapport, interviewers varied the order of interview questions but asked each interviewee all questions on guide. Interviews began with oral informed consent as per McGill University Research Ethics Board protocol. All participants agreed to be interviewed and were made aware of their interview’s use both in publication and program guidelines. All interviews were conducted in English with the exception of two interviews conducted in Hindi. Interviewers were fluent in both English and Hindi and included a native speaker of both languages. Interview durations ranged from 45 minutes to nearly 3 hours. Handwritten field notes were written during and after the interview by two members of the data collection team and were written into longer typed field journal entries at the end of each day. The data collection team met daily to debrief each interview, improve the interview guides, and begin preliminary inductive analysis.

### Data analysis

Data analysis was inductive, meaning it drew hypotheses during the research and analysis process rather than began with a clearly testable hypothesis [19], and occurred both prospectively and retrospectively. Hypotheses generated in initial interviews were tested in subsequent interviews for similarities and differences across contexts. In addition, hypotheses generated through interviews in one city were triangulated for consistency in the other three cities. After data analysis concurrent to collection was complete, we began retrospective analysis by comparing data across all four cities, reviewing interview recordings, and assessing completed notes. Relevant interviews or interview sections were transcribed (AM transcribed all interviews and translated the two Hindi interviews). Narrative analysis was undertaken to find similar topics addressed in different ways across interviews. Recurring topics were selected and interview transcripts and recordings were reviewed again to examine for links to these topics. After grouping interviews or portions of interviews around recurring topics or themes, we reviewed the interviews again for similar and dissimilar statements around selected themes to determine a prevailing sense among physicians and to understand what may contextualize those who did not agree with the prevailing opinion or experience. Finally, data were checked against programmatic data like Xpert referral and positivity rates to examine for commonalities among physicians practicing in a particular context. Themes selected included: culture, sputum, public sector engagement, empirical treatment, resistance, adherence, diagnostic process, and sensitivity/specificity.

### Funding source

The project was funded by United States Agency for International Development (USAID) under the Challenge TB project. FIND was responsible for implementation, training, coordination, monitoring, data analysis and writing of the report in close coordination with Central TB Division, Ministry of Health and Family Welfare, Government of India.

### Ethical clearance

The qualitative study presented here was approved by the Research Institute of McGill University Health Centre on 9/12/2014 under study ID 4100 and study ref number 14-173-BMB. Regarding the broader intervention, Xpert testing for presumptive TB cases is an approved intervention under RNTCP. As such, the results presented here are our experience-sharing of the approved interventions in a programmatic setting within the existing accredited RNTCP TB diagnostic lab network. Since the observations described here are a part of implementation of approved interventions under RNTCP, separate ethical clearance was not required.

## Results

A total of 55 providers were interviewed, of which 20 practiced in the public sector, 22 practiced in the private sector, 5 practiced in trust hospitals, and 8 were RNTCP officials. Thirteen interviews were conducted in Hyderabad, 15 in Chennai, 12 in Delhi, and 15 in Kolkata.

Provider interviews revealed that in spite of clear guidance from India’s RNTCP on the need for upfront Xpert testing for pediatric presumptive TB cases, there was a diversity of perspectives on how best to use Xpert and where to place it within the ‘in practice’ pediatric diagnostics algorithm. Some physicians used Xpert as a screening tool–to rule out TB, particularly for children who have a history of contact with a TB case. Others ordered an Xpert only after a positive Mantoux tuberculin skin test and a TB suggestive chest x-ray in combination with culture or as a replacement of culture. Still others used Xpert as a test of last resort, when no other conclusive diagnosis was available or in the absence of response to empirically prescribed treatment. In addition, some physicians made it clear that not all children they diagnosed with TB were referred for Xpert testing. When clinical signs and symptoms clearly suggested TB, empirical TB treatment was prescribed.

### Xpert in the diagnostic process

In each interview, physicians reported on Xpert in their diagnostic process. Xpert use was situational and based on physicians’ perceived utility of the test, awareness of the samples it accepts, felt need for microbiological confirmation, and assessment of disease severity and probability of TB. One public sector physician explained his diagnostic process as a set of tasks. He said,

“We will first do a TST (Mantoux tuberculin skin test). Then we will do a CBC (complete blood count) and check the ESR (erythrocyte sedimentation rate) levels. Then we will look to the X-ray. After that we will find out if the child has been vaccinated or not. Then we look to see if we can do a fine needle aspiration or a tissue biopsy. Then the last thing we will do is send for a CB NAAT (cartridge based nucleic acid amplification test) test and that sample we will also send for culture testing.”

When asked about a negative Mantoux tuberculin skin test he explained,

“If it is positive then only we will consider TB and with (a TB suggestive) X-ray it is clearly TB, but those cases I can say 90% do not come to us because probably they have been diagnosed at the primary level…I use CB NAAT (cartridge based nucleic acid amplification test) when other tests are negative. If tests are positive, then everyone can start ATT (anti-TB treatment). If it is negative, then I have to solve that puzzle and then I use CB NAAT (cartridge based nucleic acid amplification test). I am not using it as screening tool. We will do all the other tests and then we’ll go for CB NAAT (cartridge based nucleic acid amplification test).”–Dr. A (Public)

This physician’s choice to use Xpert after considerable laboratory evidence of TB was shared by many. Another explained, “We do not just send samples for any cough or cold. We look at a list of symptoms. We assess whether there is a probability. Only when we are absolutely, to a certain extent, clinically certain that it could be TB do we send samples…We do not want to misuse (overuse) the test.”–Dr. B (Public) A third physician reported a tendency to use Xpert on his more puzzling patients. He explained, “Maybe we have diagnosed 1/3 of our TB positive children with Xpert. The other 2/3rds are diagnosed on an outpatient basis when it is really quite clear already that they have TB. We only send Xpert samples for those who are admitted and we have a complicated diagnosis to make.”–Dr C (Public). Another physician reported that he views his colleagues’ high level of suspicion before ordering Xpert ill-advised. He commented,

“We still do TST (Mantoux tuberculin skin test) and ESR (erythrocyte sedimentation rate) and chest x-ray and then we look. If the TST (Mantoux tuberculin skin test) is negative, we can pretty much rule out TB. If it’s positive, then we keep thinking about TB. I do know a lot of people who would start on TB treatment right there, but you would want to collaborate that. Recheck the mantoux (Mantoux tuberculin skin test), and if its positive and you can have an Xpert, then why not get the Xpert?”–Dr. D (Private).

One physician suggested that the complexity of TB’s manifestation in children require a holistic screening algorithm that accounts for multiple factors before ordering an Xpert. He explained, “It’s not a screening test. We select our patients. It’s not like we send samples for everyone… Of course we look at cough but we take the entire picture into consideration. We’d like to have our sensitivity and specificity be good too so we use the guidelines, but not always in a hard and fast way. We look to the whole picture.”–Dr. H (Private medical college)

The diversity Xpert’s location in the diagnostic trajectory and interpretation of guidelines was summed up by another physician who outlined the complex medical landscape physicians like this:

I have a problem sometimes because other doctors are not keeping themselves up to date. They don’t know about all these things. At times I get patients from general practitioners and when I write CB NAAT (cartridge based nucleic acid amplification test). Whatever I write patients will go back to their doctors who say, ‘She is a junior doctor, she doesn’t know anything, just do TST (Mantoux tuberculin skin test), chest x-ray.’ I say TST (Mantoux tuberculin skin test) and x-ray are good. Ok, they are basic tests, but this is a new test and they should prescribe CB NAAT (cartridge based nucleic acid amplification test) testing.–Dr. F (Private)

Physicians reported that they referred out admitted patients for Xpert more often due to the difficulty of collecting pediatric samples in their settings and perceived severity of the disease. One reflected on attempts to test for pediatric TB on an ambulatory basis and explained:

I’ll ask so many history questions, duration of symptoms, family history, sputum production, chest x-ray. If the x-ray shows infiltration or shadows we will treat for that and if the symptom persists we will keep looking, perhaps formatting, and if it’s a small baby then we’ll start with collecting gastric aspirate. Otherwise if they are able to bring up sputum, we will take sputum. Then with the sample we’ll do smear, Xpert, culture. Then we will see. If the child is stable, we will take the sample on an outpatient basis. There’s no need to admit the children. We don’t think admission is necessary.–Dr. G (Trust)”

### Xpert and confidence

Physicians who used Xpert often found Xpert results useful to initiate treatment, give parents confidence in their diagnosis, and motivate parents to begin and continue their child’s treatment. In addition, they suggested that with an Xpert result other physicians would not question a TB diagnosis.

A private physician suggested that Xpert offered proof of a difficult diagnosis. He noted, “(Before) if we had inconclusive results, we could start the treatment and if the child does not improve we will search for some other diagnosis. Now in most of the cases we are able to prove a microbiological diagnosis, Xpert positive so it is certainly TB. Obviously, we want to prove the diagnosis.”–Dr. J (Private) Another physician spoke of the need for certainty about starting TB treatment because treatment would remove evidence of illness. He explained, “I cannot really ever prove that another doctor was wrong once treatment has started. I will have no pathological evidence after starting treatment…It’s a real frustration, because either way we cannot prove if it was or was not TB…That’s why I try really hard to get microbiological confirmation before starting.–Dr. E (Private)" The physician’s concern for evidence to corroborate his diagnosis to other physicians was elaborated upon by one who reported that an expert result was equally important for communicating with parents. She explained:

With kids it’s really difficult to tell the parents. We can convince them to get an Xpert test because its fast, a result right away, and the results are usually quite good. The specificity is really high and so is the sensitivity…Though we have to obtaining the sample is challenging, we have a good result… The parents are taking the sample and getting the result themselves. It is done by the government, so they actually have trust in it … I tell them they can go and do it privately and it will cost several thousand rupees. They say ‘Ok we will go (to the public sector)’ and they trust its quality. There was one patient who I told could go do both and put samples in both places for cross checking but in the end, they took it to only the public sector.–Dr. F (Private)

Nonetheless certainty was not always enough to prioritize Xpert as a screening tool. A physician practicing a public hospital explained:

Isolation of mycobacterium tuberculosis in children is very difficult. Although DOTS (directly observed treatment, short-course) guidance says that we have to get more accurate and do no trials of TB drugs, we used to start a trial of anti-TB drugs. Recently with the advent of this test we have really minimized these cases. None-the-less contact history, malnutrition, weight loss, symptoms, x-ray, TST (Mantoux tuberculin skin test) positive, at least two or three these points must be favorable to prescribe an Xpert. Clinical suspicion is very pertinent.–Dr. I (Public)

### Xpert and treatment initiation

Physicians who used Xpert credited it with producing a change in their empirical treatment practices. Most attribute a reduction in the proportion of TB cases initiated on TB treatment based only on clinical symptoms. They also reported a reduction in requests for other expensive tests and procedures like CT (computed tomography) scans and bronchoscopies. Furthermore, some suggested that Xpert helped patients avoid longer hospital stays. Physicians who used Xpert early in their diagnostic process linked it to a reduction in diagnostic delay as results arrived quickly allowing them to diagnose and begin treatment the same day.

Many physicians noted a change in treatment initiation practices after using Xpert. One explained,

“My empirical treatment has gone down. We used to start empirically for every third child; we would give medicine and see. If the child had fever, cough more than three to six weeks we used to start ATT (anti-tuberculosis treatment) empirically, but now with this Xpert it has actually limited our unnecessary treatment. And it was unnecessary, but once you start they have to take for 6 months.”–Dr. E (Private)

Another pediatrician suggested that Xpert put an end to his empirical treatment, but felt that its key effect was a reduction in time to from TB presumption to treatment. He explained:

The revolution from Xpert is time. It is 24 hours.…Now we have stopped empirical treatment because we know we can get the information quickly. Before we would have started empirical therapy for 8 weeks, waited until the culture comes, if it doesn’t grow then we will stop. Now we don’t start empirical at all, in fact it’s going away…Empirical therapy is a reason to get MDR TB so it should be stopped. So we’re preventing, identifying and treating resistance with Xpert.–Dr. N (Public)

A public sector physician reported that same day results and treatment initiation were key. He said, “We are very positive about it and patients are happy because they are getting the results very fast. In one day, we get the results and tell them that they can start the treatment immediately. We also get rifampicin sensitivity report, so naturally we are quite happy about it.”–Dr. I (Public) Another public sector physician explained that a fast result allows him to avoid other procedures by saying “When we get the diagnosis. I know what I am dealing with and we can manage it… If I don’t get a positive result, in six weeks’ time indirectly how many things will I have done?…I may intubate this child and all, so in that way diagnosing give a completely different result. In seven days, a child is back at home.”–Dr. Q (Public) A privately practicing physician noted,

“(Xpert) is an addition to our armory and gives us the rifampicin report. There are a few cases where we’ve really been surprised to find a positive result in Xpert, when we’ve gotten nothing from all the other tests. In the same way, there have been cases where the Xpert has come negative but the sputum has come positive for AFB (acid fast bacilli). Still, the most important thing is the speed at which we get the results.”–D. H (Private)

For many a positive result sped up treatment initiation while a negative result did not necessarily mean that physicians would not eventually treat for TB. One explained,

Any suspected case of TB where you need to start patient you also need to get a confirmation. In 6 out of 10 patients we will have positive Xpert results. For the other four we will have gotten a negative Xpert, so we have ordered a CT (computed tomography) and based on those reports or even if CT (computed tomography) is normal but all the features are showing TB then we treat it…We start treatment for the 6 who are TB positive on Xpert on the day of the result but the other 4 will not start that same day as the Xpert comes negative. Then we keep going on with other investigations.—Dr. J (Private)

Not only did sampled pediatricians consider Xpert an addition to their toolkit, some reported that it reduced their need to request expensive imaging. One said, “So if we get some positive evidence of TB in the form of symptoms, TST (Mantoux tuberculin skin test) or chest x-ray then we don’t go for CT (computed tomography) scans or bronchoscopies any more, we just get a gastric aspirate and use the CB NAAT (cartridge based nucleic acid amplification test). If it’s positive we can avoid these scans so we can cut the cost to patients by almost 75%.”–Dr F (Private) In sum our results suggest that pediatricians report Xpert access as helping them reduce empirical treatment as well as other costly imaging.

### Xpert and drug resistance

Physicians reported that using upfront Xpert testing for presumptive pediatric TB cases, raised their awareness about drug resistant TB among children. Several reported identifying resistant TB in patients they would not have presumed resistance. In one case, a physician used Xpert to rule out drug resistance in a patient he would have empirically initiated on second line anti-TB treatment. Several suggested that Xpert allowed them to practice good antibiotic stewardship and do their part to control antibiotic resistance.

One clinician practicing in a trust hospital explained that “recently we had an MDR (multidrug-resistant TB) positive child who was picked up by Xpert. For that patient we suspected it would be tuberculosis, but we had never expected it would be MDR. How can you say it’s MDR (multidrug-resistant TB) unless they have a contact with MDR? … Otherwise we would have waited to see her not responding for 3 weeks.”—Dr. K (Trust) This pediatrician’s surprise identification of drug resistance while looking for TB was also shared by another physician. He said, “We have never had a reason to suspect rifampicin resistance as we often see treatment naïve cases but still we found one case of rifampicin resistance with Xpert.”–Dr.L (Private) A third pediatrician suggested that Xpert enabled him to start diagnosing drug resistance. He explained why he was reluctant to diagnose multidrug-resistant TB before by saying,

We have started diagnosing MDR (multidrug-resistant TB) in children after Xpert was made available. Before that, most of the patients even if they (had) resistant TB they never came for follow up and the culture report would (take) 6–8 weeks. By then the patient…would have gone to some other center because he had not gotten a definitive answer from the treating physician. You cannot start empirical MDR (multidrug-resistant TB) treatment in children as it’s too toxic so we had to wait for 6–8 weeks now we have cut down on this and begun to diagnose MDR TB (multidrug-resistant TB).–Dr. M (Private)

A different physician reported that he once used Xpert to rule out rifampicin resitance after a history of close family contact. He explained:

I had a patient whose brother had died of MDR-TB (multidrug-resistant TB). He was a confirmed case of MDR-TB (multidrug-resistant TB) and the child’s parents were really worried that something would happen. They kept pushing us to do something. So we did a gastric aspirate. We did bronchoscopy and we did not find any TB bacilli, but then we did an endoscopic biopsy and there we found TB bacteria. Xpert was positive as well as being rifampicin sensitive. Now this added to our confidence that we should give only first line of ATT (anti-tuberculosis treatment). Otherwise we would have ended up giving him second line drugs because it was of course likely that this patient would have gotten TB from his brother…In his case, we had seen the bacteria so we knew it was there and that it would respond.–Dr. M (Private)

Nonetheless many physicians continue to rely on culture for information about drug resistance and send samples for both Xpert and culture. One who explained why he continued use of culture by saying, “In the past one year, we have found 8 resistant cases and of those 7 were based on Xpert. We cultured them too and got more information, but in one case we had Xpert sensitive but the culture showed resistant.”–Dr. I (Public)

### Xpert and culture

Physicians most often saw the value of Xpert in its fast turnaround time as compared to culture and its ability to test a diversity of samples. Nonetheless, few reported replacing an existing TB test with Xpert. Most continued to send samples for smear microscopy and a large group continued to use culture alongside Xpert.

One said, “Xpert is so much faster than culture. It’s really a break through”—Dr. R (Public) while another echoed, “We rely more on Xpert because culture takes too long. We get a rapid result.”–Dr. F (Private) Despite enthusiasm about the speed of Xpert, culture remained standard for many physicians. Many felt that because pediatric samples were precious, they needed to send a single sample for as many tests as possible. One commented, “Usually it’s difficult to get a sample so we send it for three, smear, Xpert, and culture.”–Dr. A (Public) Another valued the culture’s more detailed drug resistance picture. He explained, “We are sending for culture because we have a precious sample. We cannot ask for a laparoscopy again and get a peptone biopsy again. Once we’ve taken that we want it to be fool proof. Though it may already be Xpert positive, we also want to know about culture and what it is resistant too.”–Dr. H (Private Medical College) A public sector physician recounted that he had largely replaced the use of culture with Xpert. He explained:

“It goes without saying that Xpert is better than smear. Before we were using culture because… as pathologists and microbiologists a culture is gold standard. Even now if we have some doubt we send for culture, but if we have a clear clinical picture and a positive Xpert we no longer send for culture testing. We are doing every case on Xpert but not culture.”–Dr. S (Public)

As this physician’s initial remark makes clear, many physicians felt that Xpert might replace smear, but Xpert was unlikely to replace culture.

This perspective on smear was not uniform. Many physicians continued to use smear and positive smear, culture and Xpert results equally. Others had never used or discontinued ordering smears after access to Xpert. One physician explained, “We send (smears) for all cases. We would send smears anyway. Now we send for Xpert too and we take the result from whichever comes positive. If we get Xpert positive but smear negative, we treat. If we get smear positive and Xpert negative, we treat. Now we have something else extra.”–Dr. J (Public) Another, however, noted that over time he came to consider Xpert as more sensitive. He said, “We send for smear to our own labs here too. We always send for both, direct microscopy and Xpert. For us I am not sure why but even the AFB negative ones are coming Xpert positive. We have kept Xpert above AFB and we use that result as the better one. We are convinced that this is the way of the future.”–Dr. M (Private) As this physician made clear, it was more likely that physicians reported learning about Xpert through use than through product profile data. Despite reported changes in the use of smears and cultures, no pediatrician reported a change in his or her use of TST (Mantoux tuberculin skin test) or X-ray after access to Xpert.

### Xpert and samples

Difficulty obtaining pediatric TB samples may explain the ubiquity of TST (Mantoux tuberculin skin test) and X-ray. Many physicians reported a hesitation to rely on pediatric sputum samples. As one private physician explained, “Somehow, I don’t know, kids they don’t cough out and I have not been satisfied by sputum. I have not sent it, at least in my opinion. I see very small kids and if I tell them to cough out they cough superficially.” The same physician continued to discuss her reliance on TST (Mantoux tuberculin skin test) and x-ray as in part a result of the difficulty of collecting samples for children. She said,

We have to do gastric lavage which sometimes parents object to, ‘why are you putting a tube? Why not take some blood test.’…At present for fusion or empyema or some abscesses we can send. Otherwise sputum I am not comfortable. I get so many lung cases but I don’t send them. I am not comfortable and I make a diagnosis with x-ray only; x-ray, TST (Mantoux tuberculin skin test), ESR (erythrocyte sedimentation rate), whatever routine we have.–Dr. T (Private)

Her hesitance to preform a gastric lavage or bronchoalveolar lavage (BAL) was shared by others. A physician quoted above reported hesitance to collect gastric aspirates on an outpatient basis and others used Xpert exclusively on children admitted to the hospital. One explained, “A key challenge I have is that only after four days of admission I do a BAL (bronchoalveolar lavage) and then on the fifth day I get the result. I usually admit on Monday’s so that means the nurse will do the GA (gastric aspirate) or BAL (bronchoalveolar lavage) on Thursday.” She continued, “Parent’s do not always like the procedure but I do it a little later by then they have built a confidence on us.”—Dr. B (Public)

Pediatricians also reported confusion about the minimum sample volume necessary for Xpert. While explaining his criteria to select patients for Xpert, one pediatrician added, “We still do smears for fine needle aspirations because the sample is so small so we have to do smear. When we have more volume we send for Xpert.” Dr. Q (Public) Another wondered, “How big does a sample need to be? Our fine needle samples are really small, surely Xpert cannot be used for them.” Dr. S (Public) Sample collection was particularly complicated in cities where few physicians preformed BAL (bronchoalveolar lavage) on children. We understood why one particular physician seemed to send so many samples when he said, “I am the only person here who does BAL (bronchoalveolar lavage), so many other pediatricians, if they want to access Xpert, refer children to me to do BAL (bronchoalveolar lavage). Sometimes they will have already tried with sputum and could not get a good sample. I do a BAL (bronchoalveolar lavage) and send for Xpert but probably the treating physician will be someone else.”-Dr. O (Private).

A pathologist explained that sample collection was an additional cost to patients and often required referral. She said,

“I am pathologist and we have to use a ZN (Ziehl-Neelsen) stain smear. We cannot do without it. We have to use that because not all of our patients can get the sample to the center, so we cannot send. Even though they are given the test free of cost, still they are reluctant. Because we are pathologists we can at least take the sample and send it across, but other pediatricians are at a loss. They know that they will have to send the patient to a pathologist to take the sample. And that is a bit of a problem for them.”–Dr. U (Public Medical College)

### Public private interaction around Xpert

Physicians in the private sector who otherwise had no engagement with the public sector used the project Xpert labs located in public institutions. They had a high level of confidence in the quality of the result due to a strong confidence in the test itself and the staff at public laboratories. Pediatricians frequently reported that rapid turnaround time was key to their accessing a public sector machine. Several expressed surprise on receiving Xpert test results so quickly. They also found the availability of positive Xpert results from public sector lab useful when referring children for free-of-cost RNTCP treatment. One physician explained his concern for credibility and speed by saying,

I send even my patients who could easily pay 2000 rupees to the RNTCP Xpert because of the reliability. It’s not the money, it’s the reliability. I have to base my treatment on a report that they are going to give me. What good is the money if I don’t get a reliable report right away. The patient trusts what we say so even if they don’t really want to take a sample to the government I can convince them. See, it matters if it’s expensive and if it is reliable, because the patient trusts me so I have to be eligible for that trust.–Dr. O (Private)

Another private physician explained that the report rather than its origin was important. He said, “If you got a positive result it doesn’t matter how you got it (regarding sending to the public sector).”—Dr. D (Private) Another physician passionately explained that public sector medical training contributed to her willingness to access a public sector test. She said: “I have myself studied and done my post-graduation in a government institute, so why will I not send to them for free testing. I cannot say that government set ups are not good. I have done my education from a government hospital and for my child’s education would prefer a government institution…If I say I do not trust the government set up I am saying that I don’t trust myself.”–Dr. E (Private)

## Discussion

In spite of the major recent progresses in the development of TB diagnostics and guidance on their application specifically in context of Pediatric TB and EP-TB, under-diagnosis of childhood TB remains a major problem [20, 21]. Globally, a major gap exists between the estimated burden and notification of pediatric TB cases and available tools, such as Xpert, are often not implemented or not widely used even if they are available [5, 22]. The study was undertaken to better understand the perceptions and concerns around the use of Xpert from the side of the treating physicians that contribute to this important implementation gap.

Provider interviews identified the high level of diversity in practices for diagnosis of TB in children, even in the presence of clear evidence-based guidance. Considerable variety was observed with respect to placement for Xpert in the diagnostic process across different settings. This diversity was heavily influenced by the existing/past practices of the physicians. Many physicians continue to diagnose pediatric TB with reliance on Mantoux tuberculin skin test and radiology and used Xpert only for differential diagnosis in confusing cases. This is a notable divergence from WHO and RNTCP policy which recommends upfront Xpert testing for all pediatric presumptive TB patients. Given this diversity of prevalent practices, additional work needs to be done to improve provider literacy and further disseminate the evidence on newer diagnostics. Only when each pediatric presumptive TB patient is offered upfront Xpert testing a more synchronized pediatric TB case management, same day TB diagnosis, and access to prompt and accurate TB treatment can be guaranteed. Locating Xpert at the end in the diagnostic process or placing too many restrictions on the criteria of patients who can access the test will limit its impact significantly.

It was further observed that providers who used Xpert observed notable effects in their practice, particularly a reduction of empirical treatment for pediatric TB cases. Providers reported being aided significantly by a rapid Xpert test result as a tool to provide evidence for TB treatment and encourage better adherence among their patients. Provider’s appreciation of the rapid same day availability of test results was a recurrent and transversal theme through the interviews. Not only was Xpert considered a vast improvement on the several-week reporting time of culture, but the same day results provided under the current project initiative by email and/or short messaging services to providers’ mobiles, allowed physicians to start treatment on the same day thereby reducing diagnostic and treatment delays which are common in pediatric cases [23]. The providers found the 2-hours’ time to result for Xpert acceptable over the rapid turnaround of smear [15 minutes) given the gains in limiting inter-reader variation, the higher test sensitivity and the additional information provided on resistance.

Sample collection procedures required for getting quality specimen in children and limited sample volume were challenges reported by physicians and may explain their placing Xpert later in diagnostic algorithms than national guidance. Physicians varied in their choice of sample types sent to Xpert and sample collection method. Though most did not consider sample collections methods like fine needle aspiration cytology or BAL (bronchoalveolar lavage) invasive, they often tried to diagnose TB without collecting a sample. In addition, when volume of collected sample was limited physicians often preferred culture over Xpert and smear. Hesitance to preform pediatric TB sample collection techniques may explain the continued use of TST (Mantoux tuberculin skin test) and X-ray as screening tools and presents a challenge that must be addressed to continue increasing microbiologically confirmed TB diagnoses in children.

The interviews suggest that making Xpert available is important, but paying attention to the overall diagnostic turnaround time including specimen transportation, same day testing and ensuring timely result delivery is crucial to increasing uptake[24]. With longer turnaround times, it is unclear if Xpert will achieve a similar impact on routine practices and be able to effect empirical treatment as significantly as these physicians most often received a result within 24 hours. Nonetheless, this finding aligns new data in South Africa that suggests Xpert may have contributed to a reduction in empirical treatment and suggests that improved access to Xpert might help reduce non-standard practices like the ones reported here [25].

Physicians reported thinking differently about drug resistance after having access to Xpert testing. They have in many cases become more aware of the possibility of drug resistance among children. Those who received a rifampicin-resistant TB result in a child without history of contact with a DR-TB patient or previous TB treatment reported increased reliance on Xpert testing overall. This result suggests that access to Xpert and prompt results has surprised physicians by diagnosing resistance in patients for whom they did not suspect resistance. Physicians who had this experience reported that it altered their long-held views about MDR-TB risk in children. By finding such cases Xpert access may be a lever for behavior change. Clear policy and simplified access to Xpert, complemented by initiatives to improve provider awareness that will help physicians find more drug resistance even while using Xpert in idiosyncratic ways, may encourage early Xpert use.

Physicians felt more confident in the specificity of the test than its sensitivity. This is in line with available evidence on sensitivity and specificity of Xpert [7]. Physicians were concerned by what they called a high proportion of false negatives and most reported putting between half to one quarter of Xpert negative patients on anti-TB treatment anyway. This continuing skepticism of the test is made clearer by the high number of physicians who continued to send samples for smear and/or culture alongside Xpert. In the future, rapid diagnostic tests with higher sensitivity than Xpert may efficiently address many of these provider concerns.

This study is limited to physicians who engaged under the ongoing pediatric project and have referred presumptive pediatric TB patients for Xpert testing. Findings may not be representative of all providers in these cities, particularly those who have either not been mapped or, despite of various advocacy initiatives, not engaged this initiative. Further, our findings may not be applicable to physicians serving rural populations. In addition, over-reporting adherence to best practices is always possible during in-depth interviews, but correcting for this bias would only strengthen the claims here. Additional qualitative and quantitative studies of pediatric TB pathways and family interviews are important methods to collect this data from another perspective. Finally, our focus on providers primarily serving pediatric a population may leave a whole set of physicians who are families’ first resort for care out of the Xpert access paradigm. Research on general practitioners and their treatment of children is meager. Additional work is needed. Nonetheless, these results provide useful insights into the diversity of pediatric TB care practices.

## Conclusion

Access to free and rapid Xpert testing for all presumptive pediatric TB patients has had multiple positive effects on pediatricians’ diagnosis and treatment of TB. It has important effects on speed of diagnosis, empirical treatment, and awareness of drug resistance among TB treatment naive children. It is crucial to make fast high-quality Xpert services available, ‘upfront’ to all presumptive pediatric TB patients. In addition, our study shows that access to public sector Xpert machines may be an important way to encourage Public-Private integration and facilitate the movement of patients from the private to public sector for anti-TB treatment. Though there are still significant barriers to uptake of Xpert, namely a high threshold of suspicion before prescribing the test that locates it later in the clinical workup than currently recommended, these barriers can be overcome by increased access, intensive advocacy efforts and improved provider literacy, backed by efficient systems to ensure rapid overall diagnostic turnaround time.

Our study suggests that education and good policy must be in the context of high quality services at low cost. The data suggests that both turnaround time and affordability led physicians to refer their first patients for Xpert and only after they began to see Xpert corroborate their clinical findings or provide unexpected additional information about resistance did their general practice of care change. Behavior change in TB control is crucial for reducing diagnostic delay and improving quality of care but our study shows that education is ineffective as a lever of behavior change without accessible reliable, rapid, and high quality testing to go with it. In addition, our data shows that access to FOC, fast, and reliable testing has had effects on empirical diagnostic practices that education and policy alone were unable to alter.

Results also indicate that pediatricians are ready to engage the public sector for free Xpert services provided that they receive rapid results and a high quality of testing can be maintained. Our study showed diversity of Xpert utilization strategies across different providers’ despite the easy availability of rapid and free Xpert testing. The degree of diversity in TB diagnostic approaches in children reported here highlights the urgent need for a concerted effort to standardize diagnostic practices across different settings to speed the positive effects of Xpert MTB/RIF on pediatric TB care in India.
